# The abundant extrachromosomal DNA content of the *Spiroplasma citri *GII3-3X genome

**DOI:** 10.1186/1471-2164-9-195

**Published:** 2008-04-28

**Authors:** Colette Saillard, Patricia Carle, Sybille Duret-Nurbel, Raphaël Henri, Nabil Killiny, Sébastien Carrère, Jérome Gouzy, Joseph-Marie Bové, Joël Renaudin, Xavier Foissac

**Affiliations:** 1 Université Victor Ségalen Bordeaux 2, UMR 1090 Génomique Diversité Pouvoir Pathogène, BP 81, F-33883 Villenave d'Ornon, France; 2 INRA, UMR 1090 Génomique Diversité Pouvoir Pathogène, BP 81, F-33883 Villenave d'Ornon, France; 3 INRA/CNRS, Laboratoire Interactions Plantes Micro-organismes UMR441/2594, F-31320 Castanet Tolosan, France

## Abstract

**Background:**

*Spiroplama citri*, the causal agent of citrus stubborn disease, is a bacterium of the class *Mollicutes *and is transmitted by phloem-feeding leafhopper vectors. In order to characterize candidate genes potentially involved in spiroplasma transmission and pathogenicity, the genome of *S. citri *strain GII3-3X is currently being deciphered.

**Results:**

Assembling 20,000 sequencing reads generated seven circular contigs, none of which fit the 1.8 Mb chromosome map or carried chromosomal markers. These contigs correspond to seven plasmids: pSci1 to pSci6, with sizes ranging from 12.9 to 35.3 kbp and pSciA of 7.8 kbp. Plasmids pSci were detected as multiple copies in strain GII3-3X. Plasmid copy numbers of pSci1-6, as deduced from sequencing coverage, were estimated at 10 to 14 copies per spiroplasma cell, representing 1.6 Mb of extrachromosomal DNA. Genes encoding proteins of the TrsE-TraE, Mob, TraD-TraG, and Soj-ParA protein families were predicted in most of the pSci sequences, in addition to members of 14 protein families of unknown function. Plasmid pSci6 encodes protein P32, a marker of insect transmissibility. Plasmids pSci1-5 code for eight different *S. citri *adhesion-related proteins (ScARPs) that are homologous to the previously described protein P89 and the *S. kunkelii *SkARP1. Conserved signal peptides and C-terminal transmembrane alpha helices were predicted in all ScARPs. The predicted surface-exposed N-terminal region possesses the following elements: (i) 6 to 8 repeats of 39 to 42 amino acids each (sarpin repeats), (ii) a central conserved region of 330 amino acids followed by (iii) a more variable domain of about 110 amino acids. The C-terminus, predicted to be cytoplasmic, consists of a 27 amino acid stretch enriched in arginine and lysine (KR) and an optional 23 amino acid stretch enriched in lysine, aspartate and glutamate (KDE). Plasmids pSci mainly present a linear increase of cumulative GC skew except in regions presenting conserved hairpin structures.

**Conclusion:**

The genome of *S. citri *GII3-3X is characterized by abundant extrachromosomal elements. The pSci plasmids could not only be vertically inherited but also horizontally transmitted, as they encode proteins usually involved in DNA element partitioning and cell to cell DNA transfer. Because plasmids pSci1-5 encode surface proteins of the ScARP family and pSci6 was recently shown to confer insect transmissibility, diversity and abundance of *S. citri *plasmids may essentially aid the rapid adaptation of *S. citri *to more efficient transmission by different insect vectors and to various plant hosts.

## Background

*S. citri *was originally cultured from leaves of sweet orange trees affected with stubborn disease [1,2]. This phloem-restricted mollicute was later confirmed as the aetiological agent of stubborn disease of citrus and brittle root disease of horse radish [3,4]. *S. citri *is naturally transmitted to a wide range of host plants in a propagative manner by sap-feeding leafhopper vectors [5]. The successful transmission of *S. citri *by its leafhopper vector relies on the ability of the spiroplasmas to cross the gut epithelium and the salivary gland barriers [6-9]. During the insect invasion process, spiroplasmal surface proteins play a key role. Proteolysis of the *S. citri *membrane protein P89 was associated with a decrease of the adhesion of spiroplasmas to the insect vector cells [10]. Spiralin, the major lipoprotein of *S. citri *membranes, which functions as a lectin that interacts with insect glycoproteins, was shown to be required for efficient insect transmission [11,12]. Inability to achieve the complete transmission cycle within the insect vector has also been reported for some *S. citri *strains propagated *in planta *or *in vitro *[13,14]. Proteomes of such *S. citri *strains, specifically lack the membrane associated, hydrophilic P32- and P89-related proteins [14]. Protein P89, later named SARP1, is encoded by the *arp*1 gene located on the pBJS-O plasmid of *S. citri *BR3-3X [15,16]. An homologous protein SkARP1 is encoded by plasmid pSKU146 in *S. kunkelii *[17].

The genetic bases of the interaction of *S. citri *with its plant and insect hosts have been investigated with the molecular tools available for *S. citri *transformation, mutagenesis and complementation [18-22]. Using random and directed mutagenesis, fructose import was identified as one of the major determinants of *S. citri *pathogenicity [23,24]. In contrast, *S. citri *mutants unable to import glucose through the phosphotransferase system were not affected in insect transmission nor in multiplication and symptoms induction in plants [25,26]. Mutants deficient in insect transmissibility were also produced by transposon mediated mutagenesis. Disruption of the P-type ATPase resulted in a non-vectored phenotype [23], whereas disruption of the solute binding protein of a putative glucose ABC-transporter led to a reduced level of transmission [27]. To allow comprehensive, functional studies of the interaction between *S. citri *and its hosts, the genome of the insect-transmissible and triply cloned *S. citri *strain GII3-3X is currently being deciphered. Since many *S. citri *strains have been shown to carry native plasmids [28-30], we first looked for the presence of extrachromosomal molecules in genome assembly data.

## Results

### Plasmid assembly and general properties of plasmids

*S. citri *genome assembly revealed seven contigs with borders consisting of 0.3 to 2.6 kbp identical direct-repeats and presenting miniBAC insert borders organized head to tail in contigs. These contigs were also linked to themselves by sequence pairing (*i.e*. sequences of plasmid insert borders). In addition, they did not carry any sequencing reads from chromosome-specific libraries. Such properties are indicative of circular, extrachromosomal molecules. Comparison of the six largest contig sequences revealed a mosaic structure made of blocks with sequence similarities higher than 80%. These circular contigs were assigned the names pSci1 (12.9 kbp) to pSci6 (35.3 kbp), whereas the smallest one displaying no nucleotide similarity to the others was named pSciA. Plasmids pSci1-6 had G+C contents ranging from 25.6 to 29%, close to that of chromosomal DNA (26.2%), while pSciA had a lower G+C content of 21.3% (Table 1). Most of the pScis were fully cloned as 15–25 kbp miniBAC inserts. The restriction map of overlapping miniBAC inserts matched perfectly those predicted from contig sequences, demonstrating the reliability of the final contig assembly. As shown in Table 1, 28% of the miniBAC inserts corresponded to pSci fragments, suggesting their abundance in the spiroplasma cells. Relative coverage of the circular and chromosomal contigs in the largest plasmid library obtained by unbiased mechanical breakage of *S. citri *total DNA, was used to estimate the number of plasmid copies (Table 1). Since 11.5 border reads per kbp were obtained from pSci1, *i.e*. about 12 times more than from chromosomal contigs, the copy number of pSci1 was therefore estimated to be 12. As indicated in Table 1, the numbers of copies ranged from 2.5 in the case of pSciA to 14 copies for pSci5. Considering the sizes of the pSci plasmids, the amount of plasmid DNA would account for about 47% of the total DNA of *S. citri *GII3-3X cells.

**Table 1:** pSci plasmids properties and number of copies deduced from library coverage

Replicon	Size (kbp)	GC %	MiniBAC reads	Shotgun reads	Shotgun reads per kbp	Copies per chromosome	Total DNA (kbp)
Chromosome	1820	26.2	951	1741	0.96	1	1820
pSci1	12.9	28.7	6	150	11.5	12	154.8
pSci2	14.4	29	26	141	9.8	10.2	146.9
pSci3	19.3	27.8	185	232	12	12.5	241.3
pSci4	20.2	28.6	101	224	11.1	11.6	234.3
pSci5	27.8	27	50	372	13.4	14	389.2
pSci6	35.3	25.6	2	407	11.5	12	423.6
pSciA	7.8	21.3	0	19	2.4	2.5	19.5

Total pScis (% of total)			370 (28%)	1545 (47%)		74.8	1609.6 (47%)

### Genetic content of the pSci plasmids

**Figure 1:**
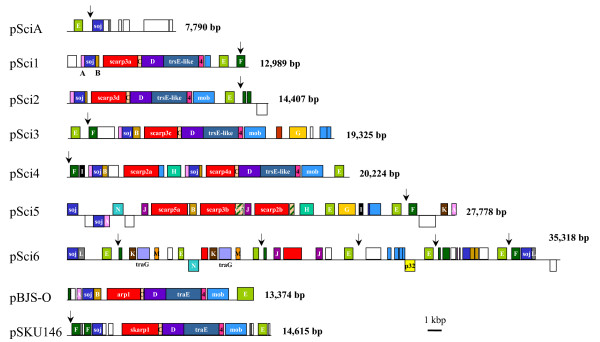
**Genetic content of *S. citri *strain GII3-3X: pScis and related plasmids pBJS-O and pSKU146**. Circular plasmids are represented by linear maps to facilitate comparisons. Conserved CDS are represented in coloured boxes. Letters A to N indicate the PA to PN paralog families. TraG, TrsE and Soj belong to TraG-TraD, TraE and parA protein families respectively. Arrows indicate the positions of IRR shown on Fig. 4 and Fig. 5.

Within a total of 138 kbp, we identified and annotated 136 coding sequences (CDS) shown in Fig. 1. In all the pSci plasmids, most of the predicted CDS were encoded by the same strand. Putative functions could be assigned to 25% of the gene products, the majority of which displayed high homology with *soj-parA*, *trsE*-*traE*, *mob *and *traG*, respectively involved in DNA partitioning (*soj*) and transfer (*trsE*-*traE*, *mob *and *traG*). One complete *mob *CDS was detected in pSci2-4, whereas the *mob *sequences detected in pSci1 and pSci5-6 encoded only truncated products. Two copies of the CDS *traG *were identified in pSci6 but not in pSci1-5. Seven CDS of pSci1-4 encoded membrane-bound TrsE and Mob proteins predicted to possess two N-terminal transmembrane domains and an ATP/GTP-binding site motif A (P-loop). A VirB4 region was detected in TrsE, while a VirD4/TraG region was predicted in Mob and TraG proteins, suggesting these CDS products are involved in a type IV secretory pathway. Eight predicted gene products encoded by plasmids pSci1-5 displayed significant similarity with the *S. citri *BR3-3X adhesion related protein P89/SARP1 and were assigned to the ScARP family (*S. citri *Adhesion Related Proteins). Two additional truncated ScARP CDS were detected in pSci6. Plasmid pSci6 encodes the P32 protein, which had been previously associated with insect transmissibility [14]. It also contains 16 truncated parts of various CDS, including disrupted copies of *soj *and *mob*. Sixty seven plasmid CDS encoded hypothetical proteins which could be grouped into 14 *S. citri *specific paralog families (PA to PN). CDS encoding PE were present in all plasmids, whereas other paralogous hypothetical protein families were only represented in some of the pSci plasmids. PC and PD were encoded by pSci1-4, whereas PJ and PN were only found in pSci5-6 and PG in pSci3 and pSci5. PH was encoded by pSci4-5. A CDS, located downstream of *trsE*-like genes of pSci1-4, shared homology with ORF4 of *S. citri *SpV1 plectrovirus [31]. Six paralog families were predicted to possess signal peptides (PC, PD, PG, PH, PI and PK), three of which have 7 (PD) and 1 (PC and PK) additional transmembrane domains. A total of nineteen orphan CDS were detected in the various pScis. As shown in Fig. 1, plasmids pSci1 and pSci2 had a genetic organization very similar to that of *S. citri *pBJS-O and *S. kunkelii *pSKU146 [15,17]. It must be noted that none of the plasmid CDS were homologous to Rep proteins involved in the replication of large plasmids of Gram+ bacteria [32]. It is noteworthy that pSci plasmids do not share sequence similarities with plasmids of phytoplasmas [33-35] that inhabit the same ecological niches as *S. citri*.

### The ScARP protein family

**Figure 2:**
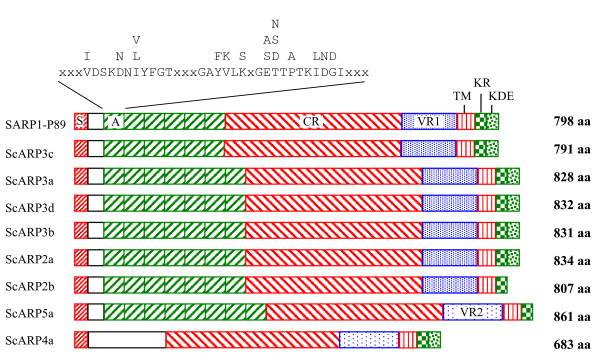
**Comparison of ScARPs domains organization**. S and TM represent putative signal peptides and hydrophobic transmembrane domains, CR and VR mean conserved and variable regions, KR and KDE are Lysine-Arginine and Lysine-Aspartate-Glutamate rich domains respectively. The amino acid sequence of the repetitive element A (sarpin repeat) and the variations observed are given above the figure.

Eight CDS were homologous to the previously characterized SARP1 of *S. citri *BR3-3X [10,16]. Based on their similarities to SARP1, ScARPs were classified in four additional families, namely ScARP2 to ScARP5 families. Plasmid pSci1-3 encoded respectively ScARP3a, ScARP3d and ScARP3c, whereas pSci4 and pSci5 carried respectively two and three *Scarp *genes, namely *scarp*2a and *scarp*4a for pSci4 and *scarp*5a, *scarp*3b and *scarp*2b for pSci5. In pSci6, two C-terminal truncated CDS of 157 and 429 amino acids were respectively 55% and 28% identical to SARP1. The sequence identities between the eight ScARPs encoded by pSci plasmids and SARP1 ranged from 40 to 77% (Table 2). ScARP2a is 83% identical to ScARP2b and both ScARP2s were found 74 to 78% identical to ScARP3s. ScARP3a, 3b, 3c and 3d shared 75 to 78% sequence identity. ScARP4a shared 35 to 53% identical amino acids with other ScARPs, whereas sequence identity of ScARP5a with the other ScARPs ranged from 50 to 62%. All *S. citri *ScARP sequences were 43 to 56% identical to the *S. kunkelii *SkARP1 (Table 2) [17]. Sizes of ScARPs ranged from 683 amino acids for ScARP4a to 861 amino acids for ScARP5a. Multiple alignment of ScARPs indicated that they possess conserved structural features (Fig. 2) [see Additional file 1]. ScARPs have a highly conserved signal peptide predicted to be cleaved after alanine 23 and a hydrophobic transmembrane alpha helix located close to the C-terminus. According to the topology prediction, ScARPs are expected to be anchored into the spiroplasma membrane with a short cytoplasmic C-terminal domain and a large surface-exposed hydrophilic domain. Except for ScARP4a, the N-terminal region of the hydrophilic part of ScARPs has 6 to 8 repetitions of a 39–42 amino acids domain previously designated sarpin repeats [16] with consensus sequence presented on Fig. 2. A central conserved region (CR) of about 340 amino acids, 64% identical among ScARPs, is located upstream of a 110 amino acid-long variable region (VR). ScARP2a, 2b, 3a, 3b, 3c and 3d share a similar VR1 sequence, while ScARP4a and 5a have a nearly identical VR2 sequence. The short ScARP C-terminus, presumably cytoplasmic, consists of a 20 amino acid stretch enriched in arginine and lysine (KR) and an optional 23 amino acid stretch enriched in lysine, aspartate and glutamate (KDE).

**Table 2:** Sequence similarity between ScARP proteins

	amino acids identity (%)									
Protein	1	2a	2b	3a	3b	3c	3d	4a	5a	sk1
SARP1	100	74	73	73	73	77	70	40	51	55
ScARP2a		100	83	77	77	78	78	41	58	56
ScARP2b			100	74	75	76	77	39	62	54
ScARP3a				100	78	75	77	35	53	54
ScARP3b					100	76	75	53	54	56
ScARP3c						100	75	44	54	55
ScARP3d							100	41	55	53
ScARP4a								100	50	44
ScARP5a									100	43
SkARP1										100

### Plasmid detection

**Figure 3:**
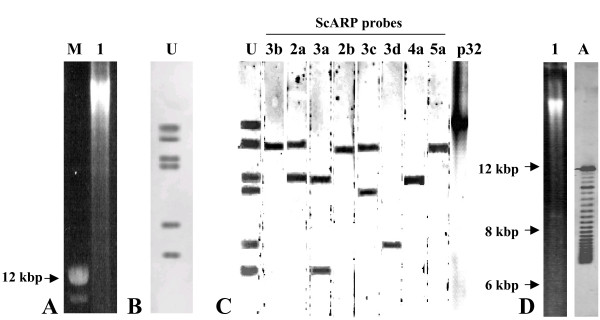
**Southern blotting hybridization of undigested *S. citri *GII3-3X total DNA**. Plasmids were separated from chromosomal DNA by long electrophoresis on 0.5% agarose gels (A, B, C) or classical 1% agarose gel electrophoresis (D) and stained with ethidium bromide (A, D track 1). Probes used for Southern blotting hybridization (C and D track A) are indicated above tracks. U, pSci1-6 universal probe; A, pSciA specific probe; M, DNA ladder. Membrane strips of a preparative gel were used for Fig. 3C hybridizations.

In the experiment of Fig. 3, the six large plasmids were separated from chromosomal DNA by a 60 hrs-long agarose gel electrophoresis (Fig. 3A, track 1) and detected by hybridization with a sequence common to all 6 pSci1-6 plasmids (probe U, Table 3). As shown on Fig. 3B, probe U strongly hybridized with 6 extrachromosomal DNA molecules distinct from the chromosomal DNA, which did not hybridize (Fig. 3A and 3B). To assign a specific contig sequence to each of the six extrachromosomal hybridizing molecules, probes specific to the various *scarp *genes were used. As shown on Fig. 3C, the 3b, 2b and 5a probes only hybridized to the second largest plasmid, therefore corresponding to pSci5. The 4a probe recognized a single band assigned to pSci4, and the 3d probe hybridized to the second molecule in size, hence corresponding to pSci2. In the same way, the *p32 *probe hybridized to the largest molecule, assigned to pSci6. However, due to high homology between *scarp *genes, probes 2a, 3c and 3a designed to respectively detect plasmids pSci4, pSci3 and pSci1, hybridized with more than one molecule (Fig. 3C). For example, probe 3c hybridized to both pSci3 and pSci4. Nevertheless, as none of the probes gave identical hybridization patterns, a pSci sequence could be assigned to each one of the hybridizing molecules, and electrophoretic migrations were in full agreement with plasmid sizes.

**Table 3:** Primer sequences that define fragments amplified by PCR and used as hybridization probes.

Primers	Nucleotide sequence (5'-3')	Accession no.	Probe's name
ScARP-2a-F	AAATATTAGTGGTGATATTTATAGAA	AJ969072	2a
ScARP-2a-R	TTATAGATTCTATGTCATCGC	AJ969072	
			
ScARP-2b-F	CAATATTAGTGGTTCTATTTATATAA	AJ969073	2b
ScARP-2b-R	CTGCAAGGGAATTTATTTGTG	AJ969073	
			
ScARP-3a-F	TTTTATACCAAAGGATATTGTTTCTT	AJ969069	3a
ScARP-3a-R	TGCAAGAGATTTAACATCTA	AJ969069	
			
ScARP-3b-F	CGGAGTTAATGATAAAGTTCAATCAA	AJ969073	3b
ScARP-3b-R	TACAAGCGAGTTTATTTGCC	AJ969073	
			
ScARP-3c-F	CGGAGTTAGTGATAAAGTTAATGCAA	AJ969071	3c
ScARP-3c-R	AGTTATTGAAATAATATCAC	AJ969071	
			
ScARP-3d-F	GGGATAAGTAATAATGCTTATTCAC	AJ969070	3d
ScARP-3d-R	CAAAAACTAGGTAATTAACATTAAC	AJ969070	
			
ScARP-4a-F	CTAAAAAAGATGTTACAACTGG	AJ969072	4a
ScARP-4a-R	ACGCATTCCAGCGTTAGACC	AJ969072	
			
ScARP-5a-F	AAATATTAATGTCTCTTCTTTAAAAA	AJ969073	5a
ScARP-5a-R	CACAAAGACACCATTACTTG	AJ969073	
			
32F1-	TAACGAATTAAATCATTCTAATAGC	AJ969074	p32
32R	TAGTTCCGGCTTGCTCACCA	AJ969074	
			
Rep-F	GCTAATAAATTAACTCGCAA	AJ969070	U
Rep-R	TTTTCCATTTTTAACTCCCGC	AJ969070	
			
pSciA-F	CCAAAAATCTAGGGCGACGA	AJ967634	A
PSciA-R	TATTGAATTGTCCCACTGCT	AJ967634	

As shown in Fig. 3D, probe A designed on pSciA, hybridized with several DNA bands from the total undigested genomic DNA (track A). The fastest and slowest migrating bands should be the covalently circular and the open circular forms of pSciA (7.8 kbp). Many pSciA supercoiled forms displaying intermediate migration were also detected by the A probe, indicating that pSciA is a highly supercoiled plasmid.

### Secondary structures and GC skew of pSci plasmids

**Figure 4:**
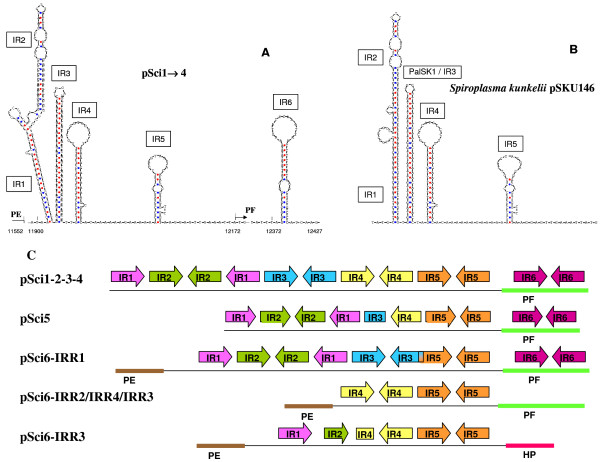
**Comparison of hairpin secondary structures and organization of inverted repeats on pScis**. Hairpins structures in IRR of pSci1-4 **(A)**, *S. kunkelii *pSKU146 **(B)**. Organization and distribution of IRR in pSci1-6 **(C)**. Such sequences marked as arrows in figure 1 could act as signals for replication or conjugation of the plasmid. Numbers below sequences (**A **and **B**) indicate positions in plasmids.

Theta replication of large plasmids in Gram+ bacteria depends on the presence of clusters of dnaA boxes at the replication origin [32]. However no cluster of dnaA box motifs (TTA/TTC/TC/A/TACA) [36] was found in pSci plasmids. Searches for palindromes and secondary structures which could act as signals for replication or conjugation revealed several sites. A cluster of inverted repeat sequences was identified in the four pSci1-4 plasmids. This region (IRR for Inverted Repeat Region) contains five inverted repeats, IR1 to IR5, upstream of the *pF *gene and IR6 downstream of the start codon of *pF*. The predicted secondary structures of the IRR regions of pSci1-4 are drawn in Fig. 4A. An IRR region, in which IR6 was absent was also detected in *S. kunkelii *pSKU146 (Fig. 4B). In pSci5, parts of IR3 and IR4 were lacking. IR4 was also absent in the mostly complete IRR1 region, downstream of position 3510 in pSci6 (Fig. 4C). Incomplete IRRs were also identified at five other locations on pSci6 (Fig. 4C).

**Figure 5:**
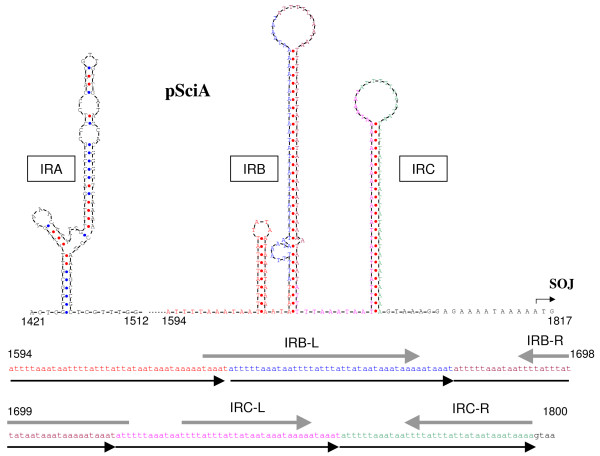
**Hairpin secondary structures and organization of inverted and direct repeats on pSciA**. This sequence marked as an arrow in figure 1 could act as signals for replication or conjugation of the plasmid. Numbers below sequences indicate positions in pSciA.

In pSciA, the non-coding region upstream of the *soj *gene contains a pair of inverted sequences able to form a hairpin (Fig. 5, IRA) reminiscent of IR1-IR2 hairpin structures of pSci1-4 (Fig. 4A). IRA is followed by a long AT stretch composed of five 41-bp direct repeats, which could also form long AT rich hairpins IRB and IRC.

**Figure 6:**
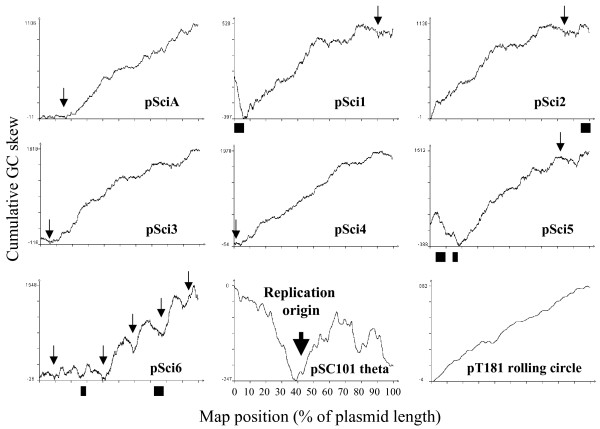
**Cumulative GC skew along pSci molecules and plasmid replicated via theta or rolling circle mechanisms**. Arrows indicate the positions of IRR shown on Fig. 4 and Fig. 5.

DNA strand compositional asymmetry can also be used to identify bi-directional replication origins by detecting a site with pronounced switch in GC skew [37]. It is the case for pSC101, which replicates through bi-directional theta replication [38]. In contrast plasmids such as pT181, that replicate through a rolling circle mechanism [39], have no such a switch in their GC skew (Fig. 6). Representation of pScis cumulative GC skew mainly indicated a regular increase for pSciA and pSci1-5, with the exception of short regions of pSci1 and pSci5, in which CDS were encoded on the opposite strand (Fig. 6). Little GC skew variation was observed in the first third of pSci6, but the rest of the replicon was characterized by an overall irregular increase of cumulative GC skew with four switches. In most cases, IRRs were located in regions of neutral GC skew preceding regions of positive GC skew.

## Discussion

Plasmids were first detected and purified in the early eighties, from *S. citri *strains isolated from naturally infected citrus, periwinkle and insect vectors collected in the South of the Mediterranean basin and south-western USA [28-30]. A small plasmid of 7.8 kbp was consistently detected in these studies. In the present work, pSciA of 7.8 kbp was characterized from strain GII3-3X and its restriction map based on nucleotide sequence data is identical to the restriction maps of the two previously reported plasmids pIJ2000 and pM41 [29,30]. Its genetic organization is described in this study, but except for the CDS encoding a Soj-like protein, no link to a specific biological function arose from the characterization of genetic content of pSciA. The functions conferred by pSciA-type plasmids therefore remain essentially unknown. The presence of plasmids larger than 10 kbp is common in *S. citri *strains. Archer and colleagues [29] isolated from *S. citri *strain SP4.6as two large plasmids of 14.8 and 35.4 kbp, similar in size to those of pSci2 (14.4 kbp) and pSci6 (35.3 kbp). It was recently shown that *S. citri *BR3-3X harbours the 13.3 kbp pBJS-O [15], the genetic map of which is highly similar to pSci2. Ranhand and colleagues [28] detected 4 different plasmids with sizes ranging from 4.1 to 26.1 MDa (6.5 to 41 kbp approximately) in all *S. citri *strains analyzed. They estimated that these extrachromosomal elements accounted for 12% of the total spiroplasma DNA. In the triply cloned *S. citri *strain GII3-3X, we estimated the plasmid content to represent nearly 50% of the total DNA, and to consist of 7 different plasmids. The presence of 7 plasmids in GII3-3X, a triply cloned strain, is consistent with the co-existence of these 7 plasmids in each spiroplasma cell. The copy numbers of plasmids pSci1-6 are not correlated with plasmid size and appear to be kept in the range of 10–14 copies per cell in the spiroplasma population. The copy numbers of pSciA could have been underestimated as the intensity of hybridization signal seems to indicate it is more abundant than calculated from sequence coverage. Two properties might explain a reduced occurrence of pSciA inserts in plasmid libraries: the very low G+C content of pSciA (21.3%) and a lower sensitivity to mechanical shearing due to its high degree of supercoiling. High copy numbers and stable maintenance of the pSci plasmids suggest efficient mechanisms for plasmid replication and partitioning. It also means that pSci plasmids are compatible and that the mechanisms of their partitioning in daughter cells are specific enough to prevent them from interfering with each other. Every pSci plasmid possesses at least one copy of *soj*, a gene encoding a partitioning protein of the parA/soj protein family, usually involved in the active segregation of low-copy-number plasmids [40]. The copy number of the pSci plasmids estimated in this study could have increased artificially due to *in vitro *cultivation; under natural conditions, it could be different.

In addition to being vertically inherited, pSci1-6 plasmids might be transferred between cells by conjugation, since they encode TrsE, TraG and Mob corresponding to VirB4/D4 components of the type IV secretion pathway, known to drive translocation of DNA through cytoplasmic membranes [41]. In walled bacteria, conjugation systems involve many other components that were not detected in the pSci plasmids. However, conjugation in wall-less bacteria might require only a reduced set of protein components. It should nevertheless involve other pSci CDS products, and especially relaxase, which is necessary for plasmid DNA binding and cleavage, and a polytopic membrane protein equivalent to VirB6, which is necessary to help VirB4/D4 ATPases to translocate the DNA through the cytoplasmic membrane [41,42]. Proteins PD, encoded by a conserved CDS on pSci1-4 and predicted to contains 7 transmembrane segments, could play such a role. None of the pSci CDS was found to share similarities with relaxase. However, the implication of a chromosomally encoded relaxase cannot be excluded. Such a situation has been described for the conjugative plasmid RP4 of *Helicobacter pylori *[43]. As reported for the *S. citri *pBJS-O [15], pSci plasmids lack the putative oriT region predicted in the *S. kunkelii *pSKU146 [17]. The possibility that the conserved IRR regions found in all pSci plasmids act as transfer origins for conjugation should be considered. Although former studies have reported the existence of genetic exchanges via a conjugation-like process in *S. citri *[44], conjugal transfer of pSci plasmids remains to be documented.

The mechanism of pSci replication is not known. Circular plasmids replicate by 3 general mechanisms, namely (i) unidirectional or bi-directional theta type, (ii) bi-directional strand displacement, and (iii) unidirectional rolling circle (RC) [32]. For pSci plasmids, the lack of switch in cumulative GC skew is indicative of an unidirectional replication mechanism. RC replication of pScis is unlikely because pScis are much larger than rolling circle replicating plasmids, which also are usually less than 10 kbp in size [45]. In addition, no CDS with similarities to rep protein of RC replication has been identified in pSci plasmids. The theta replication mode has been described in gram-negative as well as in gram-positive bacteria [32]. In Gram positive bacteria, from which *Mollicutes *originate, unidirectional theta replication has been reported for plasmids of the streptococcal/enterococcal Inc18 group such as the 26.5 kbp pAMbeta1 [46]. Their replication requires a plasmid-encoded Rep protein and the host DNA polymerase I, and is initiated from an origin located downstream of the Rep protein gene [47-49]. Such an origin of replication as well as a Rep protein have not yet been identified in pSci plasmids.

The regulatory mechanism that maintains multiple copies of pScis extrachromosomally, could help plasmid encoded determinants to better escape the *S. citri *chromosome instability known to occur during prolonged propagation in host plants [50,51]. We recently showed that ScARPs and P32 were absent from non-transmissible strains of *S. citri *such as strain 44, which also lack all pSci plasmids [14,52]. Transfer of pSci6 into *S. citri *strain 44 confers to the spiroplasmas the ability to cross the salivary gland barrier, a necessary step for insect transmission [53]. Plasmid pSci6 does not encode full length ScARPs. ScARPs, which are expected to be involved in *S. citri *adhesion to insect cells [10,16], could participate at a different stage of the insect colonization [9]. The high number of *scarp *genes raise the question of their function. The high diversity of ScARPs certainly reflects an important diversifying selection pressure exerted on these proteins. Are these different ScARPs required for *S. citri *interaction with different insect vector cell types? Or do the different ScARPs confer the ability to interact with the three leafhopper species that are known *S. citri *vectors in the Mediterranean region [54-56], where the GII3 strain of *S. citri *was originally isolated? It is interesting to notice that the Mediterranean GII3-3X strain possesses more ScARPs than does the BR3-3X strain [15] which was isolated in USA, where only *Circulifer tenellus *is known to be present. Even though diversity and abundance of *S. citri *plasmids certainly reflect its adaptation to its complex life cycle and ecological niche, the biological role of pSci plasmids remains to be further established.

## Methods

### Spiroplasma strain and cultivation

*S. citri *strain GII3 was originally isolated from the leafhopper *Circulifer haematoceps *collected in Morocco in 1980 [57]. A triply cloned strain was further produced by plating on SP4 medium and one of the clones was further propagated as GII3-3X. Spiroplasmas were grown at 32°C in SP4 medium [58].

### Sequencing and assembly

Sequencing data were produced following a chromosome map-based approach and classical shotgun strategy completed by end sequencing of inserts from a miniBAC library. Ten libraries were produced from *Apa*I and *Bss*HII overlapping large DNA fragments covering the spiroplasma chromosome. Fragments were separated by PFGE according to standard procedure, eluted from agarose gels, agarase treated and ethanol precipitated. Chromosome specific libraries were constructed in pBluescript (Stratagene, La Jolla, California, USA) after partial *Sau*3A digestion of purified chromosomal fragments. About 6,000 reads produced on ABI-prism 377XL were obtained from this map-based phase of the project. *S. citri *total DNA was purified according to Marmur's method [59] and mechanically sheared to construct two plasmid libraries. A first pSMART library of 4,000 clones with 3–4 kbp inserts (prepared by Amplicon Express, Pullman, Washington, USA) and a second pBluescript library of 2,400 clones with 1–3 kbp inserts were produced. A miniBAC library with inserts of 15–25 kbp was prepared by cloning *Sau*3A partially digested total DNA of *S. citri *in pECBAC1. Inserts were end-sequenced on ABI-prism and MEGABACE capillary sequencers.

Assembly and editing of 20,000 sequencing reads were performed with the phred-phrap-consed package [60-62]. Incorrect assembly of repeated sequences were detected by phrap, due to abnormally long distance between insert extremities, for instance, those exceeding 4 kbp for plasmid inserts. These DNA regions were assembled separately and completed by primer walking. The resulting consensus sequences were introduced back in the general assembly to resolve repeated regions and restore a normal scaffold. This strategy allowed us to resolve misassemblies due to the highly similar regions occurring in plasmids pSci1-6. The physical maps of pSci1-6 circular contigs were verified by digesting overlapping miniBAC inserts with *Eco*R1, *Eco*RV, *Hinc*II, *Hind*III and *Hpa*I. Sequences were deposited under accession numbers EMBL:AJ966734, EMBL:AJ969069, EMBL:AJ969070, EMBL:AJ969071, EMBL:AJ969072, EMBL:AJ969075, EMBL:AJ969076.

### Annotation and bioinformatic analyses

Sequence analysis and annotation were managed with the iANT (integrated Annotation Tool) web-based annotation environment developed for *Ralstonia solanacearum *genome annotation [63]. Protein-coding genes were predicted by using the FrameD program [64] trained on known *S. citri *genes and the NCBI-BLASTX program [65]. Protein motifs prediction included TMPRED for membrane spanning domains, ProDom and Prosite for conserved protein domains [66,67]. Signal peptides were predicted using SignalP 3.0 [68] and transmembrane topology predicted by TMHMM [69]. ScARPs were aligned using ClustalW [70], nucleic acid secondary structures were predicted at Mfold web server [71] and cumulative GC skew (G-C/G+C) [72] was calculated at (see Availability and requirements section for URL).

### Southern blot hybridization

*S. citri *genomic DNA was isolated using the Wizard Genomic DNA purification Kit (Promega). Whole genomic DNA was submitted during 60 h to a 0.5% agarose gel electrophoresis (0.2 volts/cm). The gel was then blotted to a positively charged nylon membranes by the alkali transfer procedure. Hybridizations with appropriate digoxigenin-11-dUTP-labelled DNA probes were carried out by using the standard method [73]. Detection of hybridized probes was achieved using anti-digoxigenin antibodies coupled to alkaline phosphatase and the fluorescent substrate HNPP (2-hydroxy-3-naphthoic acid-2'-phenylanilide phosphate) (Roche Molecular Biochemicals). Chemifluorescence was detected by using a high-resolution camera (fluor-S Multimager, Bio-Rad) and Quantity One, a dedicated software for image acquisition (Bio-Rad). Probes specific to each ScARP, probes U and pSciA were produced by PCR amplification of genomic DNA with primer pairs indicated in Table 3. Probe U consisted of a 191-bp sequence present in all *S. citri *GII3-3X plasmids pSci1-6. Probe pSciA was specific to the pSciA plasmid.

## Availability and requirements

Bioinformatics: 

## Authors' contributions

CS, PC and XF produced plasmid libraries, assembled and edited sequencing reads. CS, RH and NK carried out probe design and molecular hybridizations. SDN performed the restriction mapping of miniBAC inserts. SC and JG managed a customized iANT platform for annotation and deposited sequences at EMBL. PC, JR and XF annotated and compared pSci sequences. CS, PC and XF predicted and analyzed hairpin structures and GC skew. XF coordinated the project. XF and CS drafted the manuscript which was improved by JMB and JR.

## Supplementary Material

Additional file 1 **Multiple alignment of ScARPs**. * is for conserved amino acids, : is for partially conserved and iso-funtional amino acids and . for partially conserved amino acids. Pink and blue colours indicate positively and negatively charged amino acids respectively, red and green colours are for hydrophobic and neutral amino acids respectively. Click here for file
