# Supplementary material for: The abundant extrachromosomal DNA content of the Spiroplasma citri GII3-3X genome
Source: BMC Genomics. 2008 Apr 28;9:195. doi: 10.1186/1471-2164-9-195 (PMC2386487; doi:10.1186/1471-2164-9-195)
Supplement: Additional file 1 — Multiple alignment of ScARPs. * is for conserved amino acids, : is for partially conserved and iso-funtional amino acids and . for partially conserved amino acids. Pink and blue colours indicate positively and negatively charged amino acids respectively, red and green colours are for hydrophobic and neutral amino acids respectively. [file 1471-2164-9-195-S1.doc]

ScARP2a MKKLLSILTISTLTTSIPAPLLANTPATRTLSSNSNNDYLPLKEFKNISGDIYRMTIDSKDNIYFS-------------------------------------------LFANGAFVLKH 77

ScARP2b MKKLLSILTISTLTTSIPAPLLANTPATRTLTSNSNNEYVPIKEFNNISGSIYIMTIDSKDNIYFG-------------------------------------------TLA-GAFVLKQ 76

ScARP1-P89 MKKLLSILTISTLTASIPAPLLAAVPLTNTLTSNSNNDYLPVKQINGVNNNINSITIDKNNNLYF--------------------------------------------GTSEGAFVLKQ 76

ScARP3a MKKLLSILTISTLTASVPAPLLAAVPLTNTLTSNSNNDYLPLKENIFIPKDIVSFTVVGKGDIYY--------------------------------------------LNID-FFVLKQ 75

ScARP3b MKKLLSILTISTLTASVPAPLLAAVPLTNTLTSNSNSAYLPVKQINGVNDKVQSITVDSKNNVYF--------------------------------------------GTNNGAYKLSA 76

ScARP3c MKKLLSILTISTLTTSIPAPLLANTPATRTLTSNSNNDYLPIKEINGVSDKVNAIAVDSKNNVYF--------------------------------------------GTNNGVFVLKQ 76

ScARP3d MKKLLSILTISTLTTSIPAPLLANTPATRTLTSNSNNEISSPIKIKGISNNAYSLAVDSKNNIYF--------------------------------------------GTKDSAYKLVA 76

ScARP4a MKKLLSILTISTLTTSIPAPLLANTPATRTLSS--------------------------------------------------------------------------------------- 33

ScARP5a MKKLLSILTISTLTASIPAPLLANTPATRTLTSNSNNDYLPIKEFKNINVSSLKMTIDSKDNIYIGTYTDVLVLKHGETTATKLDGIITNAIKYSVNSLGVDSKDNIYIAKFDGAYKLSA 120

**************:*:****** .* *.**:*.:.. . . . : ... ..

ScARP2a GETTATKIDGISNNIQ--SITIDSKDNIYFGTNNGAFVLKHGETTATKIDGISN-NIQSITIDSKDNIYFGTDNGAFVLKQGATTPTKINGINGY-INSLAVDSLQNIYFSLFDNGAFVL 193

ScARP2b GATTPTKIDGINTQIN--SLAVDSKDNIYIGTDNGAYKLSAGSNTPTKIDGINT-QINSLAVDSKDNIYFGTLAGAFVLKQGETTPTKINGINTN-VYSLVVDNSNNIYYDTNG-GVFVL 191

ScARP1-P89 GETTPTKIDLSKRFIYIHAITVDSKDNIYFGTTYGIYVLNAGSDTATKINDIDN--VVSLAVDSKDNIYIGTQT-----AYG--TSQIFQSTNG--IISLVLNWNEN------------- 172

ScARP3a GATTPTKINGINDKINDFVIAVDTKNNIYFGTTNSIYFLNNGSDTATKINDINASYITKILVDSKNNIYFMSADGAYVLKHGSTTPTKINDINASYITSIAVDSKDNIYFGTKD-GAYKL 194

ScARP3b GSDTPTKINGINNIVT--AIDVDSKNNIYFGTNNGAYKLSAGETNVTSISPLPF-FVQSITVDSKNNVYFGTNNGAYKLSAGSDTPTKINGIKGG-FVSSSFDSENNIYFGKFD-GLYKL 191

ScARP3c GATTQTKINGIDGLIY--SLAVDNSNNIYFGTNGGAYKLSAGSDTPTKINGINT-NVISVIFDSKDNIYFGTLAGALVLKHGETTPTKIDGIDG-------------------------- 167

ScARP3d GSDTPTKINGIDKKVN--SLAIDSNDNVYFGTSNAVFVLKHGATTPTKIDGING-YVVSLAVDSNDNIYFGTFTNVYLLKNGATTPTKINGISGD-IATILVDSKDNIYIATNLEGVFVL 192

ScARP4a ------------------------KKDVTTGFD-------------IKIN---------EKLNNWKEVYADGKP--------------FN----------TVDNK--YYFVVWH-GLFKT 80

ScARP5a GATTATKLDGISEGIE--CTKIDSKDNIYIGTDNGAYKLSAGSNTPTKINGINE-RILNIEIDNNNNVYFGTDSGAYKLSAGSNTPTKINGINVY-VNTIAVDSKDNIYFVTLN-CVFVL 235

. : :. . . ....:: * . . . . .*. . .:. .::* . :. ::. . : . ..

ScARP2a KQGETTATKIDGISDDIESITIDSNDNIYFGMYQGGAFVLKQGETTATKIDGISDDIESITIDSNDN--IYFGMYQGGAFVLKQGETTATKIDGISNNIQSITIDSK-DNIYFGTSRGAN 310

ScARP2b KQGETTPTKIDGISDDIQSIAIDSKDNIYFGTLAG-AFVLKQGATTPTKIDGINTQINSLAVDSKDN--IYFGTLAG-AFVLKQGETTATKIDGVSNNIQSITIDSK-DNIYFGTDNGIN 306

ScARP1-P89 -------------NSAVWSLTIDSNDNIYIGTSEG-AFVLKQGKTTPTKINGINNVVNSIAVDSNDN--IYFGTANG-AFVLKQGETTPTKIDGISGAIKSLVVDKYNNNIYIGTDNGIY 275

ScARP3a SAGSTTPTKI---DSISQKFIIDSKDNIYFGTKDG-AYKLSAGSTTPTKIDSIN-GFKSIAVDSKDN--IYFGKFDG-LYKLSAGSTTPTKIDSINVDVKSLAVDKYNNNIYIKKENGIY 306

ScARP3b SAGSDTPTKINGIDDSVYSISVDSKDNIYIGTDNG-AYKLSAGSDTPTKINGINNIVTAIDVDSKDN--IYFGKFDG-LYKLSAGSDTPTKINGISGQINSLVIDKT-KYLFCGTNNGAY 306

ScARP3c ---------------RVLSVAVDSKNNVYFGTDNG-AFVLKQGATTPAKINGINTNVYSLAVDNSNN--IYFGTNGG-AYKLVVGSTTLSKIDGINGDIISITIDKD-NSVYLSSRSVWY 267

ScARP3d KNGATRPTKIKGINMHLNTIKIDSKNNIYIGTNSG-IYLLKNGATTPTKINGISGDIATILVDSKDN--IYFGTDSG-AYKLSAGETTATKIDGINVNVNYLVFDKK-NNMYIAASNGAY 307

ScARP4a N------WKIKKFNNNAEGIKIDTVGDLLMKNFGD----LTVGFGKSWKDWTNYPSYFKSVYRWDGD-----------------GEPQTPKID---------------------SNGNIT 152

ScARP5a KQGETTATKIDGIDGYVNTIAVDSKDNIYFSSFDNGVFLLKHGATTATKIDGIQPFFSMADFKFDKNNKIYFGTSNG-VFVLKQGETTTTKIDGIDGYVWTIGIDSE-NNIYFGTSKGIN 353

. . :*: .:: : . *. * .. * . : . . *. .**:. . .. ..

ScARP2a ILQ---TVLSWTKTQSQFNLVDSTKTKTWTRNDL-LSVDGELNIDIANPNIDKVVFDNIQQPQTSKQWHINVKPEIAPRDHNLQVTFTLDGKQYTS-EITVSMQAKIDPAPPSKQENLSE 425

ScARP2b ILQ---TALSWVKQQSQFALVDSTKTQTWTRNDL-LTVDGELNIDIANPNIDKIIFDNVEQPQTNKQWKINVKPEIAPRDHNLQVTFTLDGKQYTS-EITVSMQAKIDPPAPSKQENLSE 421

ScARP1-P89 FTT---TVLDWVKQQSKFTLVDNTKKQTWTQNDL-LSVDGELNIDIANPNIDKVVFDNVQQGQTNKHWTINVKPETAPRDHNLQVMFTLEGKQYTS-EIIVSMQAKINPVVSSVKQNLSD 390

ScARP3a FTT---TVLDWTNQQSKFNLVDSTKTKTWTRNDL-LSVDGELNIDIANPNIDKVVFDNIQQPQTSKHWKINVKPETTARDHNLQVTFTLDGKQYTS-EITVSMQAKIDPPAPSKQENLSE 421

ScARP3b LIHSALTSLDWTNQQSQFNLVDSTKAMTWTRPDL-LSVDGELNIDIANPNIDKVVFDNIQQPQTSKQWHINVKPETTARDHNLQVTFTLDGKQYTS-EITVSMQAKINPAPPSKQENLSE 424

ScARP3c HQQ---NILSWVKQQSQFNLVDSTKTQTWTRNDL-LSVDRELNIDIANPNIDKVVFDNIQQPQTSKQWHINVKPEIAPRDHNLQVFFTLDGKQYTS-EIIVSMQAKIDPPAPSKQDNLSE 382

ScARP3d ILALPFPVLDWVITQSKFNLVDSTKAKTWTRPDL-LTVDGELNIDIANPNIDKVVFDNVEQPQTSKQWHINVKPEIAPRDHNLQVTFTLDGKQYTS-EITVSMQAKIDPPAPSKQENLSE 425

ScARP4a DWL---VDKTKFQQGISFALAENNKTAKWSNAGMRLTVDGELNIDIDNKNIEEVYWDGTKQKILDHKANINVKPETSEKTHKLLIKYDINGKKYTSKDIEVVMAAKIDPPAPSKQDNLSE 269

ScARP5a ILQ---TALSWVKQQSQFALVDSTKTQTWTRNDL-LTVDGELNIDIANPNIDKIIFDNVEQPQTNKQWKINVKPEIAPRDHNLQVTFTLDGKQYTS-EITVSMQAKIDPPAPSKQENLSE 468

.* *.:..* .*:. .: *:** ****** * **::: :*. :* .:: ****** : : *:* : : ::**:*** :* * * ***:* .* ::***:

ScARP2a LIKTTDLGNIFDNNDDTIFSAVNQKNHNVIDDFSQIEITKKDNNSATLTAKPDSKSYAGSVDVKYNVVSATTVDLKIDVTPTSSEAAVIKDYLFQIDTSNITNPVNTFYYASSESIITMV 545

ScARP2b LIKTTDLGNIFDNNDDTIFTAVNQKNSNVIDDFSQIEITKKDNNSATLTAKKDSKSYAGSVDITYNVVPATIVDLKIDVTPISSQTTVIKDYLFQIDTSNITNPVNTFYYASSESIITMV 541

ScARP1-P89 LIKNIDLGNIFDNNDNTIFTAVNQKNHNIIDDFSQIEIPKKDNNSATLTAKKDSKSYAGSVDVKYNVVSATTVDLKIDVTPTSSEAAVIKDYLFQIDTSNITNPVNTFYYASSESIITMD 510

ScARP3a LIKTTDLGNIFDNNDDTIFSAVNQKNHNVIDDFSQIEITKKDNNSATLTAKKDSKSYAGSVDVKYNVVSATTVDLKIDVTPTSSTATVIKDYLFQIDTSNITNPVNTFYYASSESVITMD 541

ScARP3b LIKTTDLGNIFDNNDDTIFSAVNQKNHNVIDDFSQIEITKKDNNSATLTAKKDSKSYAGSVDITYNVVPATIVDLKIDVTPISSQTTVIKDYLGQIDTSNITNPVNTFYYASSESIITMV 544

ScARP3c LIKTTDLGNIFDNNDDTIFTAVNQKNHNVIDDFSQIEITKKDNNSATLTAKPDSKSYAGSVDITYNVVPATIVDLKIDVTATSSEAAVIKDYLFQIDTSNITNPVNTFYYASSESIITMV 502

ScARP3d LIKTTDLGNIFDNNDDTIFSAVNQKNHNVIDDFSQIEITKKDNNSATLTAKKDSKSYAGSVDVKYNVVSATTVDLKIDVTPTSSQAAVIKDYLFQIDTSNITNPVNTFYYASSESIITMV 545

ScARP4a LIKTTDLGNIFDNNDDTIFSAITQKNARVVD-FSQIKITDKTDTQATLSAIEGSKSYHGSVAVTYNVASATTVDLKIDVTSSASGIQIDSDYLAQLDSSKMTNKVDTFYYSNGKSTIKIK 388

ScARP5a LIKTTDLGNIFDNNDDTIFTAVNQKNSNVIDDFSQIEITKKDNNSATLTAKKDSKSYAGSVDITYNVVPATIVDLKIDVTSSASGIQIDSDYLAQLDSSKMTNKVDTFYYSNGKSTIKIK 588

***. **********:***:*:.*** .::*.****:*..* :..***:* .**** *** :.***..** ********. :* : .*** *:*:*::** *:****:..:* *.:

ScARP2a KPTPSSVLTGVVYGCDDKWNKTSQSSNIDSTNGIKLDGSQLATIKGKYVVELS--DNLGHTNNVYLQINEKKEKEIKEYWNTDNGKHFEIWAKANGYDNIRGYSASQLNNLFADSKNWQQ 663

ScARP2b KPTPSSVLTGVVYGCDDKWNKTSQSSNIDSTNGIKLDGSQLATIKGKYVVELS--DNLGHTNNVYLQINEKKEKEIKEYWNTDNGKQFEKWAKANGHYNIRGYSASQLNNLFADSKNWQQ 659

ScARP1-P89 KPTPSSVITGVVYGCDDKWNKTSQSSNIDSTNGIKLDGSQLATINGKYVVELS--DNLGHTNNVYLQINEKKEKEIKEYWNTDNGKQFEIWAKANGYDNIRGYGASQLNNLFADSKNWQQ 628

ScARP3a KPTPSSVLTGVVYGCDDKWNKTSQSNNIDPTNGIKLDGSQLNTKEGKYVVELS--DNLGHTNNVYLQIAQ--DKKIPNYFDTDNGKHFEIWANANGYDNIRGYSASQLNNLFADSKNWQQ 657

ScARP3b KPTPSSVLTGVVYGCDDKWNKTSQSSNIDSTNGIKLDGSQLATIKGKYVVELS--DNLGHTNNVYLQINEK-KKDIKEYWNTDNGKQFEIWAKANGHYNIRGYSASQLNNLFADSKNWQQ 661

ScARP3c KPTPSSVLTGIVYGCDDKWNKTSQSSNIDSTNGIKLDGSQLATIKGKYVVELS--DNLGHTNNVYLQINEKKEKEIKEYWNTDNGKQFEIWAKANGYDNIRGYSASQLNNLFADSKNWQQ 620

ScARP3d KPTPSSVLTGVVYGCDDKWNKTSQSNNIDPTNGIKLDGSQLNTKEGKYVVELS--DNLGHTNNIYLQIAQ--DKKIPNYFDTDNGKQFEIWAKANGYDNIRGYSASQLNNLFADSKNWQQ 661

ScARP4a QPSTGNFITGIVYGCDEKWNKTSQSNNIDLTTGLEIDKGQYGSVDGRYLIELQHKDLPTHTKTIYLQISE--KQKIEHYWDTPKGKQFEIWAEDNGEKNIRGYSASQLNNLFEISAIWKQ 506

ScARP5a QPSTGNFITGVVYGCDDKWNKTSQSSNIDPTTGLEIDKGQYGSVDGRYLIELQHKDLPTHTKTIYLQISE--KQKIEHYWDTPNGKKFEEWAEDNGEKNIRGYGASQLNNLFEISAIWKQ 706

:*:....:**:*****:********.*** *.*:::* .* : .*:*::**. * **:.:**** :...:.* .*::* :**:** **: ** *****.******** * *:*

ScARP2a LAS--DSQLASVVADWFK--TNGKLSATEPLTKEQVVEQLKTQISSDIKIDKVNTNNYEKDKVSFVLNQSEFKPNDKVNITVKYNNATSEQFTLQIKD-----SKTPDNKKDGDNKFWII 774

ScARP2b LAS--DSQIASAVADWFK--TNGKLSATELLTTEQVVEQLKTQIPSDIKIDKVNTSNYEKDKVSFVLNQSEFKPNDKVNITVKYNNATADSFTLQIKD-----SKTPDNKKGGDSKLWII 770

ScARP1-P89 LAS--DSQLASAVADRVK--TSGKLSSTAPLTKEQMVEQLKTQIPSDIKIDKVNTTNYEKDKVSFVLNQSEFKPNDKVNITVKYNNATSEQFTLQIKD-----SNTPDNKKDGDSKLWII 739

ScARP3a LAS--DSQIASAVADRVK--TSGKLSSTAPLTTEQVVEQLKTQIPSDIKIDKVNTNNYEKDKVSFVLNQSEFKPNDKVNITVKYNNATSEQFTLQIKD-----SKTPDNKKDGDNKFWII 768

ScARP3b LAS--DSQLASAVADRVK--TNGKLSATEPLTKEQMVEQLKTQIPSDIKIDKVNTNNYEKDKVSFVLNQSEFKPNDKVNIAVKYNNATSEQFTLQIKD-----SNTPDNKKDGDNKFWII 772

ScARP3c LAS--DSQLASAVADRVK--TNGKLSSTAPLTKEQVVEQLKTQIPSDIKIDKVNTNNYEKDKVSFVLNQSEFKPNDKVNITVKYNNATAEQFTLQIKD-----SNTPDNKKDGDNKFWII 731

ScARP3d LAS--DSQIASAVADWFK--TNGKLSATELLTTEQVVEQLKTQIPSDIKIDKVNTSNYEKDKVSFVLNQSEFKPNDKVNITVKYNNATADSFTLQIKD-----SNTPDNKKDGDNKFWII 772

ScARP4a SLKHLDLKLDNFVVDNIKNVSQDEIYTYKTKMLASVKEQVENYVPG--VVENTDYVININNLVAGDWTTSKDVKVQAVDGSTKLLSFDSKTIPVEQKEQVTPLTSTPDNKKDGDSKLWII 624

ScARP5a SLKHLDLKLDNFVVDNIKNVSQDEIDTYKTKMLASVKEQVENYASG--VVENTDYVININNLVAGDWTTSKDVKVQAVSGSTKLLSFDSKTIPVEQKEQVTPLTSTPDNKKDGDNKFWII 824

. * :: . *.* .* :..:: : .: **::. ... :::.: :: *: . *: : *. :.* . :. :.:: *: :.******.**.*:***

ScARP2a GVVVGVLAGLGLAYLLFKKFVFDKYFLPKINKRRHDKLVEKVRKEEAEKDAQNNKKGGDE 834

ScARP2b GVVVGVLVVLGLAYLLFKKFVFDKYILKRRSRKRSRK----------------------- 807

ScARP1-P89 GVVVGVLVVLGLVYLLFKKFVFDKYFLPKINKRRHDKLVEQVKKEEAEKDAQNNK-GGEK 798

ScARP3a GVVVGVLAGLGLAYLLFKKFVFDKYFLPKINKRRHDKLVEKVRKEEAEKDAQNNKKGGDE 828

ScARP3b GVVVGVLAGLGLVYLLFKKFVFDKYFLPKINKRRHDKLVEKVRKEEAEKDAQNNK-GGDE 831

ScARP3c VVVVGVLAGLGLAYLLFKRFVFDKYFLPKINKRRHDKLVEKVRKEEAEKDAQNNKKGGDE 791

ScARP3d GVVLGVLAGLGLAYLLFKKFVFDKYFLPKINKRRHDKLVEKVRKEEAEKEAQNNKKGGDE 832

ScARP4a GVVVGVLAGLGLAYLLFKKFVFDKYFLPKINKRRHDKLVEKVRKEEAEKEEENNKGGDE- 683

ScARP5a GVVLGVLAGLGLAYLLFKKFVFDKYILKRRSRKRSRK----------------------- 861

**:***. ***.*****:******:* : .::* * .. ...:... .... ....

**Additional file 1 : Multiple alignment of ScARPs.**

* is for conserved amino acids, **:** is for partially conserved and iso-funtional amino acids and **.** for partially conserved amino acids. Pink and blue colours indicate positively and negatively charged amino acids respectively, red and green colours are for hydrophobic and neutral amino acids respectively.
